# Low levels of sex hormone-binding globulin predict an increased breast cancer risk and its underlying molecular mechanisms

**DOI:** 10.1515/biol-2022-0822

**Published:** 2024-02-08

**Authors:** Shuhang Zhao, Jiaojiao Gu, Yu Tian, Ruoyan Wang, Wentao Li

**Affiliations:** Department of Breast Surgery, Zhengzhou University People’s Hospital (Henan Provincial People’s Hospital), Zhengzhou, 450003, China; Department of Breast Surgery, Henan Provincial People’s Hospital, Zhengzhou, China

**Keywords:** sex hormone-binding globulin, breast cancer, endocrine therapy, transcriptomics, proteomics, endocrine drug-resistance

## Abstract

Sex hormone-binding globulin (SHBG) is a serum glycoprotein exhibiting the unique feature of binding sex steroids with high affinity and specificity. Over the past few decades, there have been significant breakthroughs in our understanding of the function and regulation of SHBG. The biological role of SHBG has expanded from being considered a simple sex hormone transporter to being associated with several complex physiological and pathological changes in a variety of target tissues. Many factors can affect the plasma SHBG levels, with fluctuations in circulating levels affecting the development of various diseases, such as increasing the risk of developing breast cancer. This article reviews the clinical significance of changes in circulating SHBG levels in the development of breast cancer and the possible influence of these levels on endocrine drug resistance in hormone receptor-positive breast cancer. Higher levels of plasma SHBG significantly reduce the risk of estrogen receptor-positive breast cancer, especially in postmenopausal women. Moreover, the molecular mechanisms by which SHBG affects breast cancer risk are also summarized in detail. Finally, transcriptomics and proteomics data revealed that SHBG expression in breast tissue can effectively distinguish breast cancer from normal tissue. Additionally, the association between SHBG expression levels and various classical tumor-related pathways was investigated.

## 1 Introduction

Sex hormone-binding globulin (SHBG) is a homodimer plasma glycoprotein of 90–100 kDa that is produced mainly by hepatocytes and then secreted into the blood. SHBG is encoded by a single gene located on the short arm of chromosome 17 [1]. Although circulating SHBG is synthesized mainly in the liver, its genes are also expressed in the brain, uterus, breast, ovary, placenta, prostate, and testes [2,3]. Over the years, SHBG has generally been considered only as a passive carrier protein of sex hormones with a high affinity for androgen and estrogen that combines mainly with these sex hormones in the blood to adjust their bioavailability [4,5]. However, in the past few decades, its biological role has expanded from being a transporter or storage of sex hormones to affecting a variety of physiological and pathological changes in a variety of tissues and organs [6].

Currently, the commonly used endocrine therapy drugs for treating breast cancer include selective estrogen receptor modulators (SERMs), selective estrogen receptor down-regulators (SERDs), and aromatase inhibitors (AIs). These agents are generally only used for hormone receptor-positive breast cancer [7]. The SERM, tamoxifen, has a molecular structure similar to that of estrogen and competes with estradiol for receptors in the target organ. However, as the drug does not have the biological role of estradiol, it inhibits the growth of tumor cells [8]. SERDs such as fulvestrant also have an anti-breast cancer role by inducing degradation of the estrogen receptor (ER), blocking ER transcriptional activity, and reducing the mobility of nuclear ERs [9]. AIs such as anastrozole, letrozole, and exemestane are used commonly in breast cancer treatment to inhibit the CYP450 family aromatase, block the transformation of androgens secreted by peripheral tissues into estrogen [10], and reduce the systemic estrogen level of postmenopausal patients. Today, endocrine therapy plays an important role in adjuvant postoperative therapy for early ER-positive breast cancer to prevent recurrence and reduce mortality and also in advanced metastatic ER-positive breast cancer to prolong survival, maintain quality-of-life, or delay chemotherapy intervention [11]. Estrogen antagonists are the main drug used for this purpose because estrogen dependence on cell growth in ER-positive breast cancer patients often persists after several anti-estrogen treatments. Unfortunately, a part of ER-positive breast cancer patients are insensitive to endocrine therapy due to primary and secondary drug resistance [12,13].

This article reviews the clinical significance of changes in circulating SHBG levels in the development of breast cancer and the possible influence of these levels on endocrine drug resistance in hormone receptor-positive breast cancer. Additionally, the molecular mechanisms by which SHBG affects breast cancer risk are also summarized in detail. However, there is a paucity of research on the correlation between local SHBG expression and breast cancer. By means of bioinformatics analysis, we found that SHBG expression in breast tissue can effectively distinguish breast cancer from normal tissue and investigated the association between SHBG expression levels and several tumor-related pathways.

## 2 Plasma SHBG levels are considered as biomarkers of human disease

In the past few decades, it was found that the cell membranes of many tissues express SHBG receptors [14] and that circulating SHBG molecules can enter cells. The binding of SHBG to its receptor has been shown to induce activation of cyclic adenosine monophosphate (cAMP) [15]. This intracellular signal transduction pathway is important for many biological processes, including cancer growth [16]. Recent studies have emphasized the potential value of measuring SHBG levels for assessing the risk of a variety of other diseases. Of these, a low SHBG level is associated with metabolic syndrome, obesity, insulin resistance and type 2 diabetes, hypothyroidism, hormone excess (androgen, progestin, growth hormone, and glucocorticoid), and a higher risk of cardiovascular disease and breast cancer [17,18,19,20], while conditions associated with an increased SHBG level include aging, hyperthyroidism, liver disease, human immunodeficiency virus disease, bone loss, and a higher risk of fracture [21,22]. These findings have important implications for developing preventive measures for various diseases. However, the change in SHBG level is only a biomarker, and further studies are required to establish whether SHBG is actively involved in the pathogenesis of the above-mentioned diseases. In addition, although the role of SHBG in the development of breast cancer is complex and may play different roles according to the immunohistochemical phenotype of the ER [20], observational data have shown that circulating SHBG concentration correlates negatively with the risk of postmenopausal breast cancer, especially ER-positive breast cancer [23].

The molecular characteristics of SHBG may provide a potential new target for preventive or therapeutic interventions [24]. However, it is necessary to further study its molecular mechanisms and investigate its potential therapeutic effects. As the structure and function of SHBG are clarified in greater detail, the specific downstream effects of the SHBG receptor complex in intracellular signal transduction will be worthy of further study.

## 3 Clinical significance of SHBG levels in predicting breast cancer development

Plasma SHBG levels are not only associated with a variety of benign endocrine diseases [25,26,27] but also affect the incidence of several sex hormone-dependent malignancies [28], such as low levels of circulating SHBG may increase the risk of breast, endometrial, and prostate cancer [29,30]. Perhaps because breast cancer is the most common malignancy in women and also the most common sex hormone-dependent tumor [31], SHBG and breast cancer have been significantly more studied than the latter two cancers. In addition, existing studies have focused on the morbidity of overall breast cancer, suggesting that low SHBG levels increase the risk of breast cancer compared to controls [32]. A small number of studies based on tumor receptor status have concluded that low SHBG levels increase the risk of ER-positive breast cancer more significantly than overall breast cancer [20]. Researchers tend to get more statistically significant results when studying the onset of postmenopausal breast cancer [33], whereas the correlation is often less significant in premenopausal breast cancer studies [34,35]. This review shows some recent studies involving plasma SHBG levels and breast cancer risk (Table 1).

**Table 1:** Studies on plasma SHBG levels and the incidence of breast cancer

Study	Source	Cases	Controls or total	HR or OR (95% CI)	*p*-value	References
James et al. (2011)	Germany	378	596	0.71 (0.51–1.00)	0.0400	[36]
Sieri et al. (2012)	Italy	356	1,537	0.72 (0.51–1.02)	0.0700	[32]
Fourkala et al. (2012)	UK	200	400	0.48 (0.25–0.89)	0.0220	[37]
Zhang et al. (2013)	USA	258	515	0.68 (0.44–1.04)	0.0400	[38]
Duggan et al. (2016)	USA	43^ａ^	358	0.48 (0.26–0.89)	0.0200	[39]
		102^ｂ^		0.64 (0.43–0.97)	0.0400	
Hüsing et al. (2017)	EPIC cohort*	787	1,292	0.86 (0.77–0.96)	0.0074	[34]
Dimou et al. (2019)	Greece	122,977^ｃ^	105,974	0.94 (0.90–0.98)	0.0060	[20]
		69,501^ｄ^	95,042	0.92 (0.87–0.97)	0.0030	
Arthur et al. (2020)	UK	492	120,516	0.75 (0.56–0.99)	0.0280	[40]
Watts et al. (2021)	UK Biobank	1,870	111,170	0.88 (0.83–0.94)	0.0002	[30]
Arthur et al. (2021)	UK Biobank	890	43,373^ｅ^	0.70 (0.56–0.88)	0.0010	[41]
Tin et al. (2021)	UK Biobank	2,997	133,294	0.89 (0.84–0.94)	—	[35]
Chen et al. (2022)	USA/China	—	—	0.83 (0.73–0.94)	—	[42]

*EPIC cohort: European Prospective Investigation into Cancer and Nutrition (EPIC) cohort.

^a^Breast cancer-specific mortality; ^b^All-cause mortality; ^c^Overall breast cancer; ^d^ER+ ve disease; ^e^Non-cases.

## 4 Mechanisms by which low plasma SHBG levels promote breast cancer

### 4.1 Plasma SHBG regulates free sex hormone levels

SHBG is a specific binding protein with a high affinity for sex hormones and binds androgens and estrogens with nanoscale affinity. As a consequence, SHBG acts as the main transporter and recognized regulator of sex hormones in plasma [43]. According to the free hormone hypothesis, the biological activity of sex hormones is affected by their free concentration in the circulation [44]. This provides a mechanism by which SHBG may regulate the action of sex hormones. Sex hormones that are not bound to SHBG are considered to be bioavailable and circulate freely (about 13% of the total) or are bound loosely to other proteins, such as albumin. In this way, the level of free available sex hormones in plasma is influenced greatly by the concentration of SHBG [21].

Lower levels of SHBG indicate higher levels of both free estrogen and androgen. In addition, in women, as a result of SHBG having a higher affinity for testosterone than for estradiol, a reduction in its plasma level causes the circulating androgen system to become relatively active. This results in reduced sensitivity of the hypothalamus to the luteinizing hormone (LH) pulse, leading to excessive production of the gonadotropin-releasing hormone that stimulates the pituitary gland and release of LH to promote the total level of estrogen and androgen [1]. In addition, estrogen promotes SHBG synthesis, while androgen inhibits its expression. Therefore, increased SHBG levels indicate a relative greater activation of estrogen compared to that of androgen [45]. Nevertheless, under these conditions, the absolute level of free estrogen is reduced.

### 4.2 SHBG reduces the risk of ER-positive breast cancer by downregulating free estrogen levels

Endogenous steroid hormones play a central role in the development of breast cancer, with lifetime exposure related to elevated circulating estrogen concentration. This increase is associated with recognized risk factors for breast cancer, such as an early age of menarche, late first-term delivery, childless, late menopause age, and need for hormone replacement therapy [46]. Multi-observational studies have confirmed the relationship between estrogen and androgen and the high risk of premenopausal breast cancer [47] and postmenopausal ER-positive breast cancer [48]. SHBG is one of the factors that regulate estrogen balance, and therefore, it must be considered when assessing the risk of breast cancer in elderly women. Key et al. analyzed personal data from nine prospective studies [23] that included 663 women with breast cancer and 1,765 women without breast cancer and indicated a significant correlation between higher levels of sex hormones and an increased risk of breast cancer, with increased SHBG levels related to a reduced risk of breast cancer. Other studies have also reported that not only high estrogen levels but also increased circulating testosterone levels have a significant effect on ER-positive breast cancer [49]. This finding indicates that higher androgen levels after menopause are more conducive to the conversion of estrogen [50]. Therefore, a decrease in the SHBG level leads to an increase in circulating free sex hormones, which promotes the proliferation of mammary epithelial cells and inhibits cell apoptosis, thereby preventing the destruction of precancerous cells [51] and increasing the risk of postmenopausal breast cancer, especially ER-positive breast cancer.

### 4.3 SHBG interacts with breast cancer cell membranes through receptors and affects intracellular pathways

**Figure 1:**
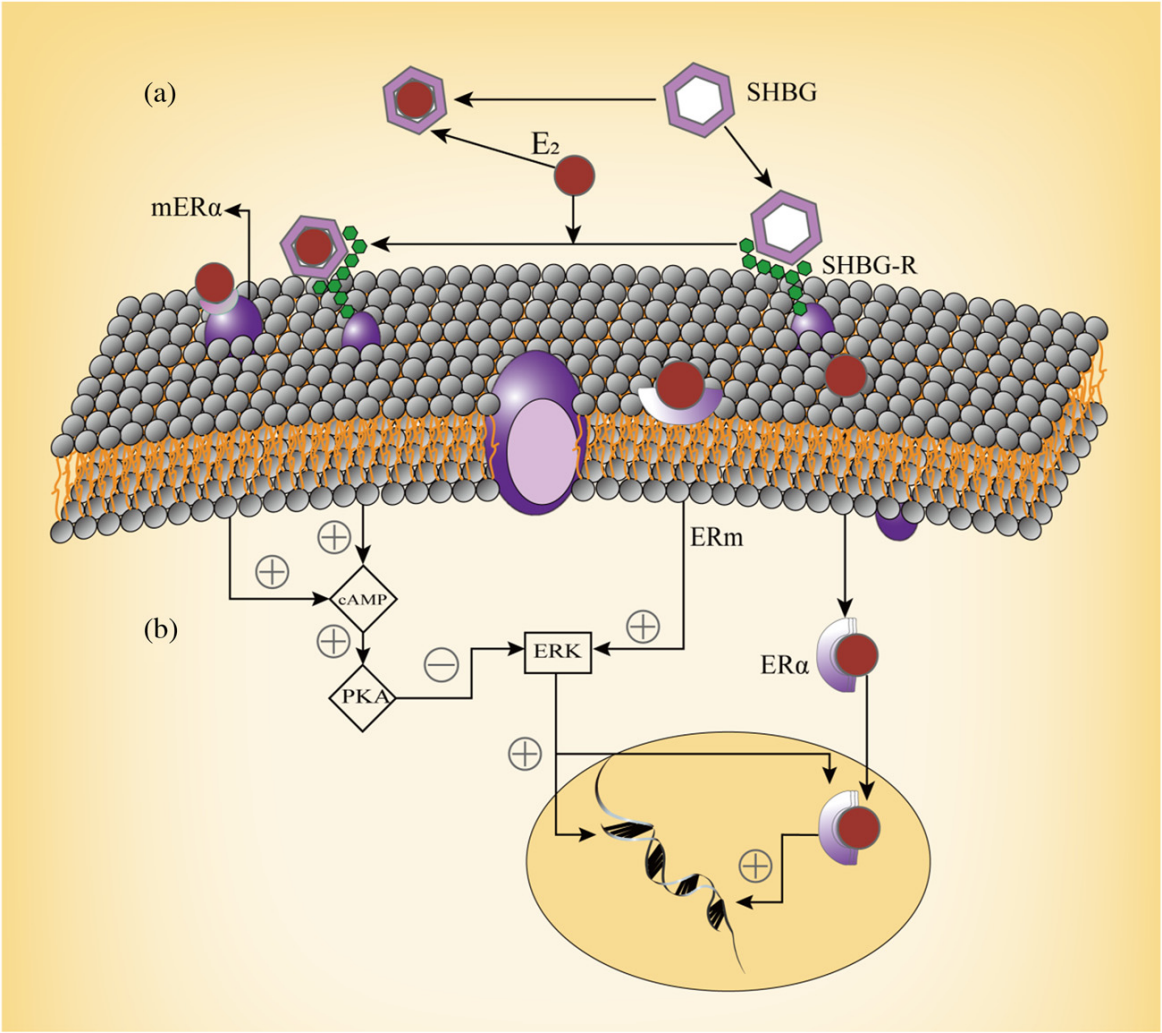
(a) SHBG interacts with the membrane through a receptor. If SHBG binds to the sex hormone first, the interaction between it and the cell will be inhibited, whereas when a macromolecule binds to the membrane first, it has the same affinity with the steroid as when in solution. (b) The binding of SHBG to the MCF-7 cell membrane results in a signaling cascade. E2, estradiol; ERm, membrane estrogen receptors; mERα, membrane-bound estrogen receptors alpha; ERα, estrogen receptor alpha. Partially prepared from previous studies [52,56].

The membrane interaction of SHBG with estrogen-dependent MCF-7 breast cancer cells is a time- and temperature-dependent highly specific binding relationship, which implies SHBG interacts with the membrane through a receptor capable of associating its extracellular macromolecular ligand with intracellular pathways [52]. The cell membrane can only bind with SHBG, which is free from steroids. If it binds to the sex hormone first, the interaction between SHBG and the cell will be inhibited, whereas when SHBG binds to the membrane first, it has the same affinity with the steroid as when in solution [53] (Figure 1). This relationship is consistent with the previous description that only free sex hormones are biologically active. The sensitivity of breast cancer cells to estrogen is closely associated with the interaction between SHBG and cell membranes. The binding site of SHBG has been characterized in ERα-positive MCF-7 cells, while no binding site has been detected on ERα-negative and estrogen-insensitive MDA-MB 231 cells [54]. In tissue samples from breast cancer patients, the proliferation rate of samples with a steroid-binding protein receptor bound with SHBG was reduced significantly compared to samples that could not bind with SHBG [55].

When SHBG binds to the MCF-7 cell membrane, it initiates a signaling cascade, leading to an increase in intracellular cAMP induced by estradiol. This increase in cAMP level and activation of its target protein, PKA, inhibits estradiol-induced activation of ERK, a member of the mitogen-activated protein kinase family that plays a critical role in regulating cell proliferation. This inhibition of ERK reduces the antiapoptotic effect induced by estradiol, thereby inhibiting the proliferation of breast cancer cells [56] (Figure 1).

### 4.4 SHBG interferes with the ability of estradiol to regulate genes in breast cancer cells

**Figure 2:**
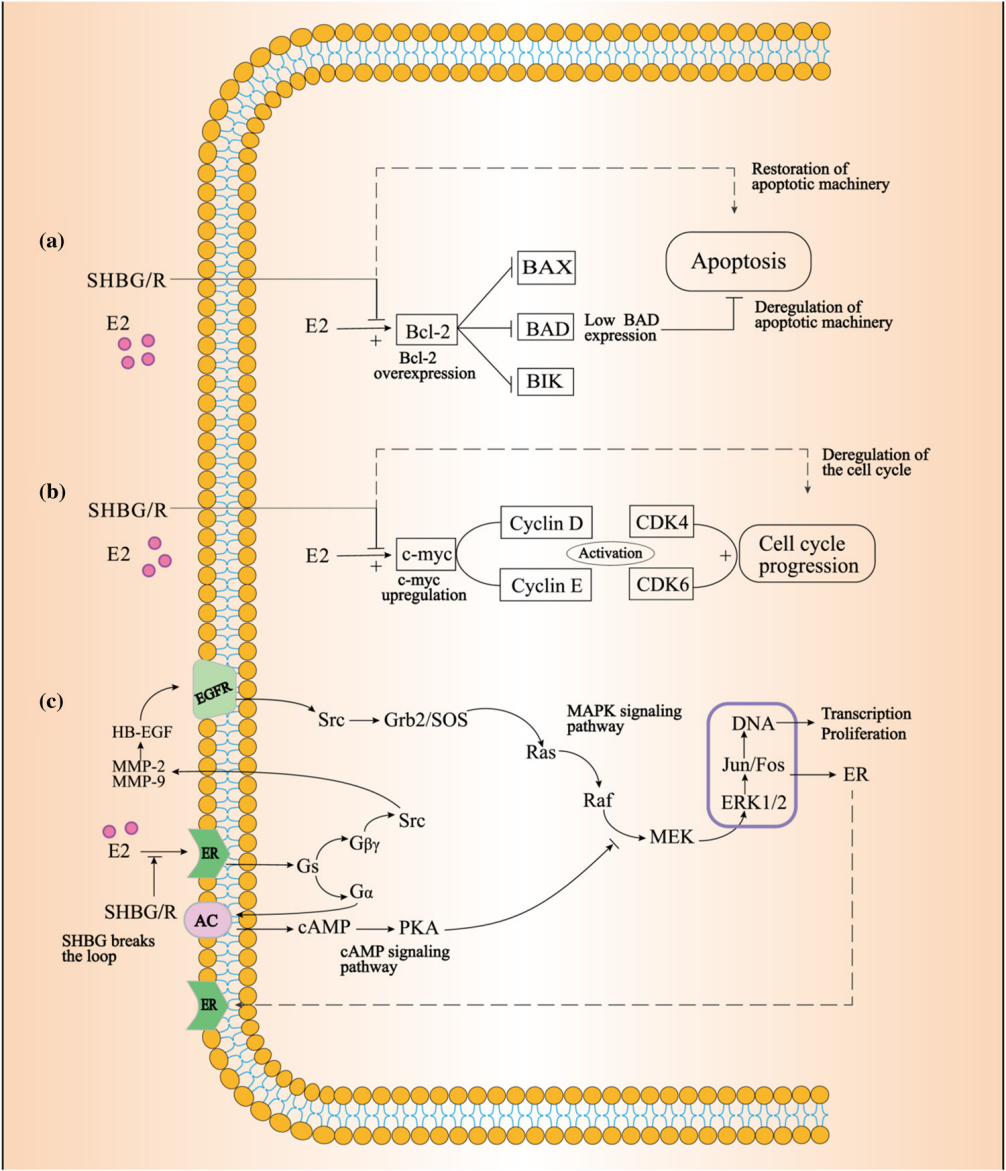
(a) Estradiol induced bcl-2 overexpression and inhibited apoptosis; SHBG interferes with estrogen, downregulates Bcl-2, and restores apoptosis. (b) Estradiol induced c-myc overexpression and promoted cell circulation; SHBG interferes with estradiol, downregulates c-myc, and inhibits DNA replication. (c) Estradiol induced EGFR overexpression, while activation of EGFR-derived signaling pathways amplified the role of estradiol; SHBG disrupts the loop connecting the two pathways by inhibiting estradiol-induced EGFR expression, thereby reducing the growth of breast cancer cells. E2, estradiol; EGFR, epidermal growth factor receptor; Src, tyrosine-protein kinase; Grb2, growth factor receptor-bound protein 2; SOS, son of sevenless; Partially prepared from KEGG database (map01522).

Several genes are affected by SHBG, including those involved in cell growth, apoptosis control, or estrogen dependence in cells [52]. For example, SHBG downregulates the proto-oncogenes, bcl-2 and c-myc, and the growth factor receptor [52]. Proto-oncogene bcl-2 expresses antiapoptotic protein and is overexpressed in breast cancer [57]. The capacity of SHBG to suppress bcl-2 expression counteracts the typical gene amplification caused by estradiol, which may be one of the mechanisms of SHBG binding to its membrane receptor and then combining with estrogen to induce recovery of apoptosis in breast cancer cells. Several factors in the cell cycle play an important role in ER-positive breast cancer. Studies have shown that ER antagonists reduce the expression of cyclin D, with continuous expression of cyclin D known to lead to anti-estrogen drug resistance [58]. In breast cancer cells, oncogene c-myc is upregulated by estradiol and then impacts several key cell cycle regulatory targets. In particular, c-myc regulates the expression and activity of cyclin D, cyclin E, CDK4, and CDK6 [59,60], thereby affecting cell cycle progression. However, SHBG inhibits estradiol-induced c-myc expression, resulting in the elimination of the amplification effect on the positive regulation of the cell cycle and growth induced by estradiol. This prevents the overexpression of cyclin, which, in turn, prevents the generation of anti-estrogen drug resistance, changes that are conducive to improving the effect of endocrine therapy. Activation of the growth factor receptor pathway is also associated closely with endocrine resistance in ER-positive breast cancer. For example, overexpression or amplification of HER2, a member of the epidermal growth factor receptor (EGFR) family, can lead to resistance to endocrine drugs [61]. EGFR overexpression may be caused by estradiol, while activation of its derived signaling pathways amplifies the role of estradiol in breast cancer [62,63]. Therefore, SHBG disrupts the loop connecting the two pathways by inhibiting estradiol-induced EGFR expression, thereby reducing the growth of breast cancer cells (Figure 2).

## 5 A decrease in SHBG levels promotes endocrine therapy resistance in ER-positive breast cancer

**Figure 3:**
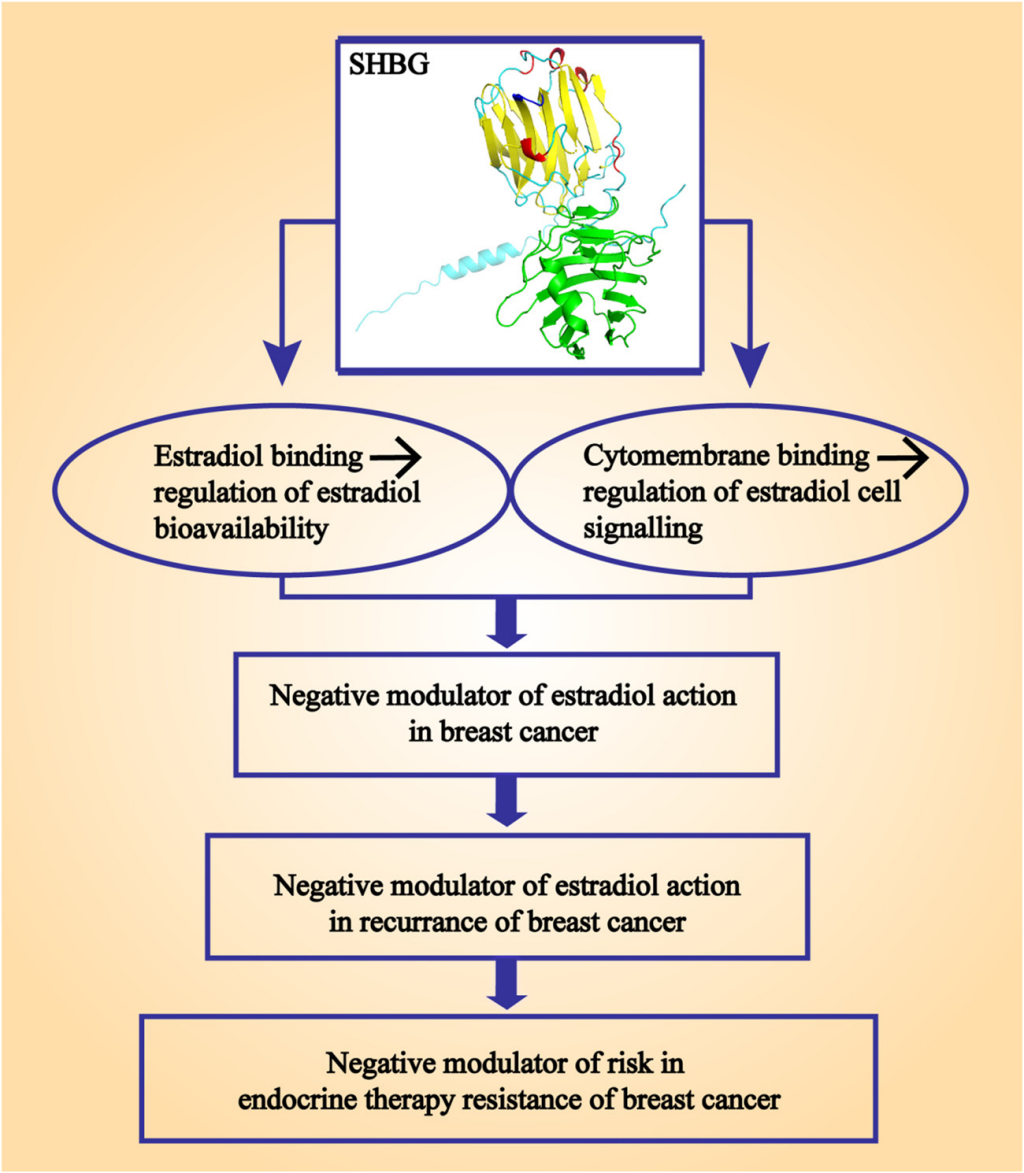
SHBG, estradiol, breast cancer, and endocrine resistance. Partially prepared from the study of Fortunati et al. [52].

Definitions of drug resistance to ER-positive breast cancer vary in international treatment guidelines, but most are based on the duration of the response to endocrine therapy or the time to recurrence after completion of endocrine therapy. For example, according to the guidelines of the European Society of Oncology (ESMO) for metastatic breast cancer [64], primary endocrine resistance can be defined as recurrence within the first 2 years of adjuvant endocrine therapy or progression in the first 6 months of first-line endocrine therapy. Secondary resistance was defined as recurrence 2 years after initiation of adjuvant endocrine therapy, recurrence within the first 12 months after completion of adjuvant therapy, or progression of metastatic breast cancer 6 months after initiation of endocrine therapy. The definition of endocrine therapy resistance provides a broader space for investigating the role of SHBG in endocrine therapy resistance. Because only the impact of SHBG on the therapeutic effect and recurrence risk of breast cancer during and after endocrine therapy needs to be analyzed, this is beneficial for clarifying the correlation between SHBG changes and endocrine therapy resistance of ER-positive breast cancer (Figure 3).

### 5.1 Decreased SHBG levels reduce the efficacy of endocrine drugs by increasing free estrogen levels

Tamoxifen is used mainly in endocrine therapy for premenopausal ER-positive breast cancer [65]. SHBG levels in healthy women increase gradually before menopause [45]. A decrease in SHBG levels not caused by sex hormone changes can lead to an increase in free estrogen and free androgen, with the increase in free androgen stimulating the release of LH to further enhance estrogen levels by blocking the negative feedback regulation of estrogen and progesterone to LH [66]. Tamoxifen works by competing with estrogen for receptors, and if more estrogen is available, tamoxifen treatment may be less effective, or at a higher dose may even lead to an increased risk of relapse after completion of treatment and withdrawal.

AI drugs are used mainly to treat postmenopausal ER-positive breast cancer [67]. After menopause, SHBG levels in healthy women decline in the short term and then increase [68]. The short-term decline in SHBG may be related to a sudden reduction in estrogen after menopause, which does not have a lasting impact on the effect of endocrine therapy. However, in the long term, if SHBG decreases too much, the levels may remain low for a long time and then rise slowly, or if SHBG decreases in postmenopausal women due to other factors, free androgens will increase. This change is more conducive to the transformation and production of estrogen, thereby reducing the effect of endocrine therapy drugs.

### 5.2 Antagonistic effects of SHBG on estrogen in ER-positive breast cancer cells help to improve endocrine therapy

The membrane initiation pathways of SHBG and estrogen function at different levels, either in the membrane or at the ERK site, ultimately inhibiting cell proliferation and inducing apoptosis, which is beneficial for improving the effect of endocrine therapy and reducing the risk of recurrence after treatment. In addition, by preventing estradiol from upregulating bcl-2, c-myc, and EGFR [52], SHBG is conducive to the recovery of the apoptosis mechanism of breast cancer cells. However, it is not conducive to excessive replication and amplification of cancer cells and also not conducive to the negative role of estrogen in the occurrence and development of breast cancer. These effects may improve the efficacy of endocrine therapy on ER-positive breast cancer and reduce the occurrence of drug resistance. In contrast, the decline in SHBG level may promote the occurrence of drug resistance.

## 6 Analyzing the molecular mechanisms of SHBG affecting breast cancer based on transcriptomics and proteomics data

Despite numerous studies on the impact of SHBG in plasma on breast cancer risk, there remains a lack of research into the effects of local SHBG expression within the breast. To further validate the predictive ability of SHBG for breast cancer risk, we analyzed SHBG expression in both normal and cancerous breast tissues using data from the Cancer Genome Atlas (TCGA) database and the Genotype Tissue Expression Project (GTEx) database (TCGA: https://portal.gdc.cancer.gov/; GTEx: https://commonfund.nih.gov/gtex). Our results were also validated by proteomic data of CPTAC samples from the UALCAN (https://ualcan.path.uab.edu) [69,70,71,72].

### 6.1 Data and methods

**Figure 4:**
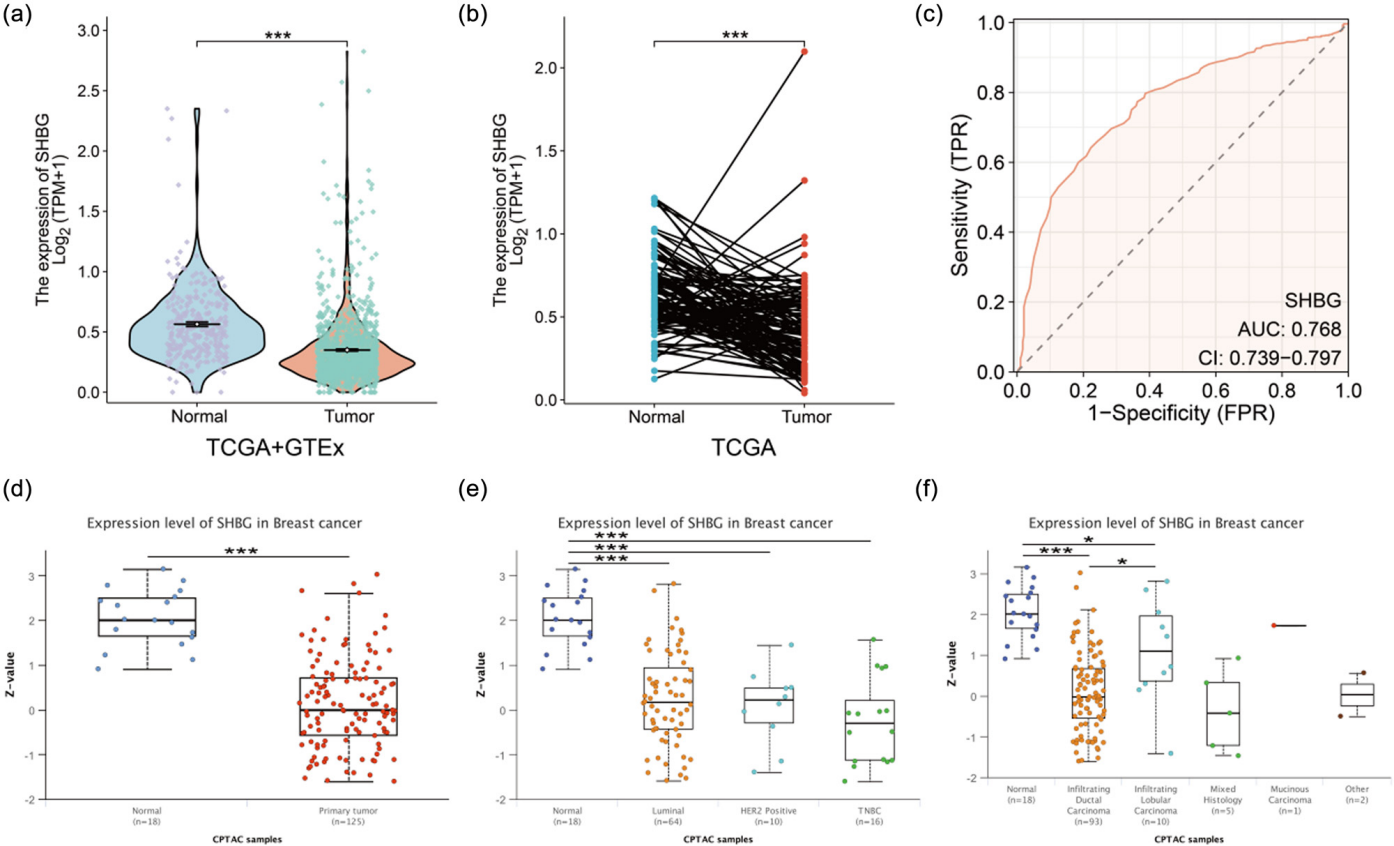
SHBG expression levels in normal breast tissue and breast tumors. (a) The expression level of SHBG in breast cancer tissue is lower than that in normal breast tissue, based on RNA sequencing data from TCGA and GTEx databases, with *p* < 0.001. (b) Differential expression of SHBG in paired samples of breast cancer patients from TCGA database. (c) The predictive efficacy of SHBG for breast cancer, by receiver operating characteristic (ROC) curve and area under curve (AUC). SHBG proteomic expression profile based on (d) sample types, (e) major subclass, and (f) tumor histology. ***, *p* < 0.001; *, *p* < 0.05.

We obtained the TPM format RNA-sequencing data of TCGA-BRCA (1,099 tumor samples; 113 para-cancer samples) and GTEx (179 normal samples) for breast cancer analysis. TCGA + GTEx samples were used for differential expression analysis by the Wilcoxon rank sum test, including 1,099 tumor samples RNA sequencing data and 292 non-tumor data (Figure 4a). A paired samples *t*-test was used for differential expression analysis, using 113 pairs of matched tumor and paracancerous data from the TCGA samples (Figure 4b). The data are analyzed using the Xiantao academic analysis tool (https://www.xiantaozi.com/products) [73], where visualization was implemented through the R (version 4.2.1), stats[4.2.1] package, car package, and ggplot 2 [3.3.6] package.

We obtained the SHBG proteomic expression profile based on sample types, major subclass, and tumor histology by using the UALCAN-CPTAC dataset (Figure 4d–f). The resulting figures were analyzed and downloaded directly online and retouched in Adobe Illustrator.

Furthermore, we performed a systematical evaluation of somatic alterations at the pathway level in breast cancer using the CPTAC dataset, which combined proteomic, whole-exome, and copy number alteration data. This involved critical pathways and genes that were previously annotated across multiple cancer types based on domain knowledge (pathway annotations from PMID: 33568653) [74,75].

### 6.2 Results

In breast cancer samples, the level of SHBG expression was considerably lower than in normal breast tissues, as indicated in Figure 4a (*p* < 0.001). Moreover, SHBG exhibited low expression in 113 matched breast cancer tissues with a *p*-value of less than 0.001, as shown in Figure 4b. In addition, SHBG expression exhibited an area under the ROC curve (AUC) of 0.768 (95% confidence interval [CI]: 0.739–0.797) for discriminating breast cancer from normal tissues, as depicted in Figure 4c, indicating its potential as a reliable predictor.

The expression level of SHBG protein in primary breast cancer is lower than that in normal tissue (Figure 4d, *p* < 0.001), regardless of breast cancer subclass (Figure 4e, all *p* < 0.001). In addition, in the results based on tumor histology, SHBG protein expression was significantly lower in infiltrating ductal carcinoma, infiltrating lobular carcinoma, and mixed histology (Figure 4f, all *p* < 0.05).

**Figure 5:**
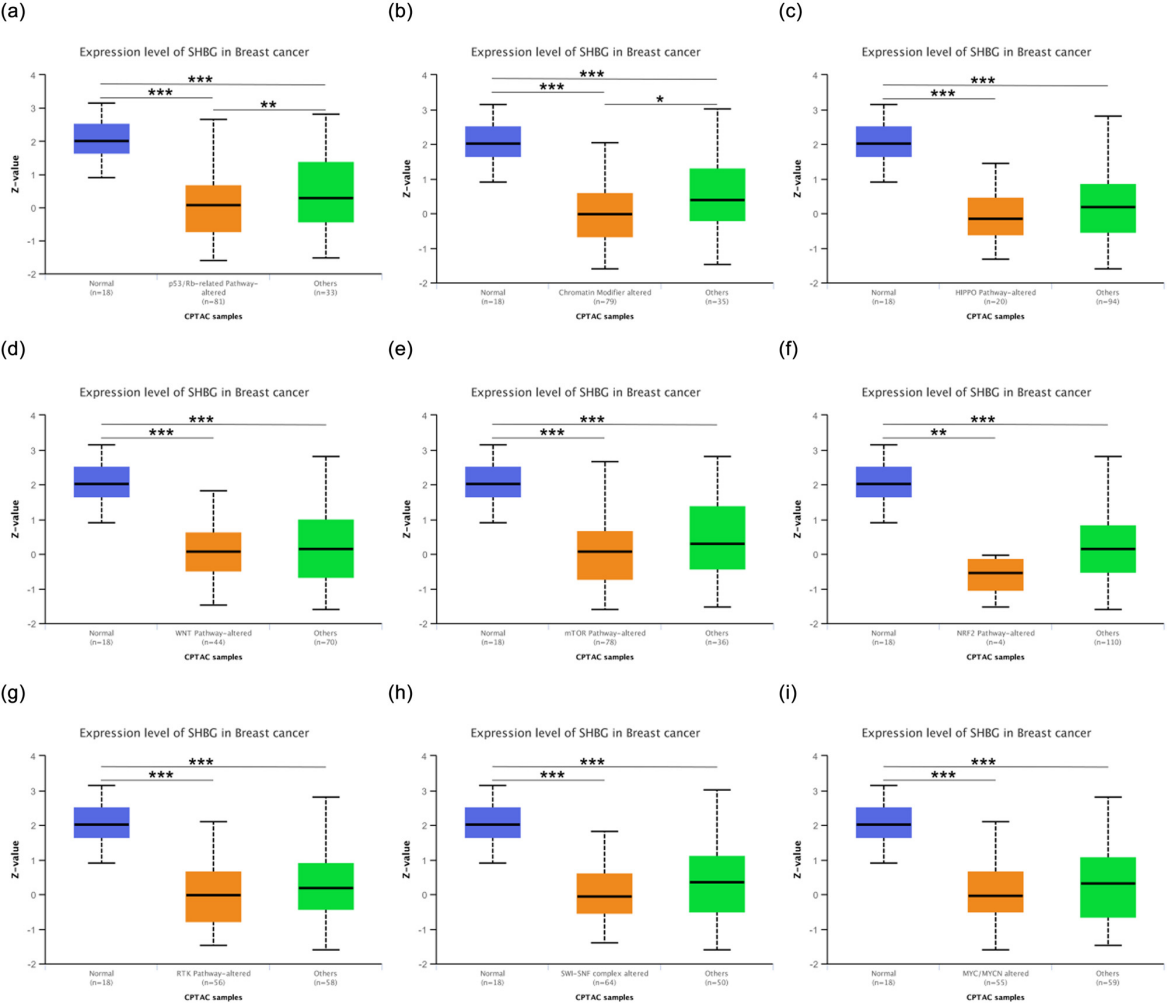
SHBG proteomic expression profile and its association with common tumor-related molecular pathways. (a–i) Pathway-level somatic alterations across tumors, involving key pathways and genes previously annotated across multiple cancer types based on domain knowledge (pathway annotations from PMID: 33568653), with all *p* value < 0.001. ***, *p* < 0.001; **, *p* < 0.01; *, *p* < 0.05.

A total of nine pathways were analyzed, including the status of the p53/Rb-related pathway, chromatin modifier, HIPPO pathway, WNT pathway, mTOR pathway, NRF2 pathway, RTK pathway, SWI-SNF complex, and MYC/MYCN. The expression of SHBG was lower in all samples with altered pathways compared to the normal control groups (Figure 5a–i; all *p* < 0.01). Meanwhile, the expression of SHBG was lower in samples with altered p53/Rb-related pathways or chromatin modifiers than in their respective corresponding samples with unaltered pathways (Figure 5a and b; *p* < 0.05). These findings suggest that the low expression of SHBG in the breast may be associated with the nine tumor-related pathways mentioned above, particularly alterations in p53/Rb-related pathway status and chromatin modifier status, thus confirming that low SHBG expression may be a risk factor for breast cancer development.

## 7 Conclusions and perspectives

Changes in human SHBG levels are influenced by many *in vivo and in vitro* factors and have been found to have a wide range of clinical implications. Many of these factors cause changes in SHBG levels and include estrogen level, age, glucose metabolism, and a diet high in fat, all of which are also generally believed to be related to the incidence of breast cancer. In addition, gene polymorphisms are also important factors affecting SHBG levels, and more in-depth studies are needed in the future [76,77]. We can exert positive effects on serum SHBG by modulating some known factors [25]. For example, (1) encourage a sensible diet, reducing fat and sugar and increasing dietary fiber intake; (2) moderately increase physical exercise [78] to relieve obesity and fatty liver [79]; and (3) control good thyroid metabolism and glucose metabolism levels.

Studies and published literature have shown that SHBG levels are related to breast cancer. Increased plasma SHBG levels can act through the following mechanisms: (1) downregulation of free estrogen levels; (2) binding to the membrane SHBG receptor and interfering with the role of estrogen in breast cancer cells; and (3) inhibiting the ability of estrogen to regulate cancer-related genes. These effects can help to fight breast cancer, improve the effect of endocrine therapy for ER-positive breast cancer, and hopefully reduce the risk of recurrence after endocrine therapy and the occurrence of drug resistance. Not only are changes in plasma SHBG clinically significant, but SHBG generated locally in the mammary gland is also more likely to produce noteworthy effects. For example, we found that the expression level of SHBG in breast cancer tissue is significantly lower than that in normal tissue through transcriptomics and proteomics analysis, and the decreased expression of SHBG in breast tissue may be associated with alterations in multiple pathways related to tumorigenesis. If it is possible to specifically increase the synthesis of SHBG by breast tissue cells in the future, this may have a significant positive effect in the treatment of ER positive breast cancer.

Unfortunately, SHBG has not shown a positive effect on hormone-receptor-negative breast cancer, such as triple-negative breast cancer, which is less incident. Meanwhile, in the few studies in premenopausal patients, although there is a trend to show positive results, these findings are often not statistically significant, which may be related to insufficient sample size and data analysis design. In order to obtain more accurate results, it is necessary to increase the data of these patients in future studies and design more reasonable data collection and statistical analysis methods. Furthermore, among the most widely utilized SERMs, tamoxifen not only exhibits competitive inhibition of estrogen but also occasionally exerts estrogen-like effects in certain tissues, such as the endometrium, bone, and heart tissues [80,81]. This particular attribute has been linked to potential resistance to endocrine therapy [82]. The authors propose that tamoxifen’s estrogen-like activity could potentially impede the beneficial impacts of SHBG in the endocrine treatment of premenopausal patients with ER-positive conditions. In other words, maintaining moderate SHBG levels may potentially mitigate the development of tamoxifen resistance arising from the estrogen-like activity of tamoxifen. Finally, research studies on the relationship between serum SHBG and breast cancer recurrence during endocrine therapy are particularly rare, and more attention is needed in the future. Further investigation is also warranted to explore the diagnostic and therapeutic potential of local SHBG expression in breast tissue.
